# Will the use of industrial robots promote transformation of export trade modes? Empirical evidence from China

**DOI:** 10.1371/journal.pone.0267135

**Published:** 2022-06-08

**Authors:** Dongmei Wang, Guangqin Li

**Affiliations:** 1 Gansu Agriculture Technology College, Lanzhou, Gansu, PR China; 2 School of International Trade & Economics, Anhui University of Finance & Economics, Bengbu, Anhui, PR China; Shandong University of Science and Technology, CHINA

## Abstract

Based on *China’s Industrial Robot and Customs* data from 2006 to 2019, this paper conducts an empirical investigation into the impact of industrial robot usage on export trade structures and its working mechanism with the popularization of industrial robots and the expansion of export trade in China. It finds that the use of industrial robots has significantly increased the proportion of general trade, reduced that of processing trade and promoted the transformation from processing-trade into general-trade, or the impact mechanism of industrial robots on export trade structure. After adopting such two methods as lag phase and tool variables to address potential endogenous problems, the results continue to be valid. In addition, the paper also discussed the regional heterogeneity effect of industrial robot trade transformation. Such evidence provides a new perspective for developing countries to explain the transformation of their export trade and are of high significance in optimizing the structure of trade patterns and promoting the transformation of export trade.

## Introduction

**Fig 1:**
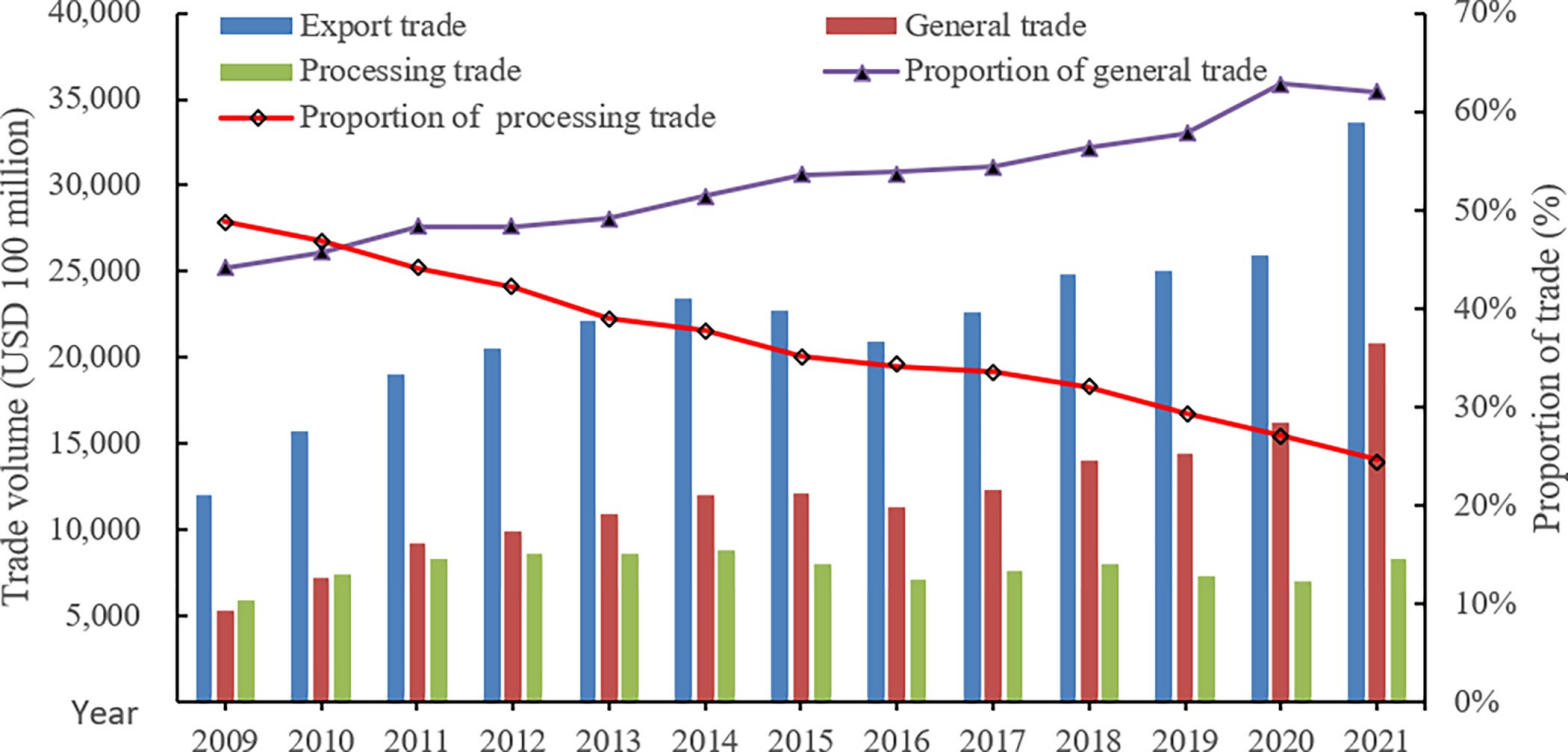
Trends in the proportion of general and processing trade in export trade from 2009 to 2021.

Since becoming an official member of the World Trade Organization on December 11, 2001, China’s export trade has made a historical leap. Fig 1 shows the trend of China’s total export trade from 2009 to 2021, where it can be concluded that China’s total export trade has been climbing steadily, from $1.2 trillion in 2009 to $3.37 trillion in 2021. The rapid development of export trade has also brought about changes in the world industrial division of labor mode. The forms of trade among countries are no longer dominated by completed products trade, while the proportion of raw materials, parts, technological patents and logistics services in trade volume is increasing. With a high level of labor endowment, a large number of export enterprises use raw materials, parts, instruments and patented brands from around the world to complete the final process of production, bringing the simultaneous development of trade scale and trade mode, which allows the characteristics of dual trade structure in China’s export trade show.

As is seen from the figure, processing trade accounted for 48.85% and general trade accounted for 44.09% of export trade in 2009. The proportion of general trade increased year by year, while that of processing trade continued to decline. In 2010, the proportion of processing trade decreased to 45.68% and that of general trade increased to 46.92%, while processing trade was still slightly higher than general trade. However, in 2011, the proportion of processing trade fell to 44%, lower than that of general trade at 48.31%. Since then, the proportion of general trade exceeded that of processing trade, rendering it China’s main form of trade. With the deep adjustment of the international competition pattern, China’s economic development has entered a new normal, and the export trade structure is in urgent need of being transformed and upgraded to cater to the new situation. The structural transformation of export trade is the transition from processing trade to general trade [1–3]. Transformation guidance for processing trade is a main focus of the report named the Nineteenth National Congress of the Communist Party of China, and also a major task for the Central Committee of the Communist Party of China since the Third Plenary Session of the Sixteenth Central Committee. The trade mode not only reflects the position of China’s export enterprises in the global value chain [4], but also affects the spillover effect of exports on technological progress, industrial upgrading and economic growth [5]. How to promote the transformation of China’s export trade mode and enhance China’s position in the division of labor in global value chains are important topics that requires urgent attention from the government and academia.

According to the existing literature, scholars who study the impact of industrial robots on the social economy, pay more attention to the relationship between the use of industrial robots and manufacturing employment, productivity, industrial upgrading, and other factors, while scholars who study export trade mode pay attention to the relationship between export trade mode and production efficiency, labor costs, financing constraints, industrial policies, economic growth, and other factors. At present, there is no research on the relationship between the use of industrial robots and export trade patterns. Therefore, our study on the impact of the use of industrial robots on export trade mode is a link and supplement to the two aspects of research, which has certain theoretical significance. On the other hand, the significance comes from the importance of exports to economic growth. Studying the impact of the industrial robot application on the structure of China’s export trade mode is helpful to find a mechanism to promote the optimization of export trade mode on micro-level, so as to provide theoretical support and countermeasures for the transformation and upgrading of export trade and the realization of the goal of *high-quality development of trade*. The above is the practical significance and potential application values of the research. Given that China has become the largest and fastest-growing country for industrial robot applications, this series of problems is particularly important.

## Literature review and theoretical hypothesis

At present, the research between the use of industrial robots and export trade mode is related to this paper, mainly in the following three aspects:

### The use of industrial robots, productivity, and the transformation of trade modes

Many studies have confirmed that the use of industrial robots can improve labor productivity and total factor productivity through capital accumulation, technological progress, management efficiency, and production stability. Graetz & Michaels analyzed a panel data of industries in 17 countries 1993–2007, and found that the application of industrial robots increased labor productivity by about 0.37 percentage points [6]. Kromann et al. using industry-level panel data for nine countries investigated the effects of automation on TFP, and found that more intensive of industrial robots has a significantly positive effect on TFP [7]. Aghion et al. believe that industrial robots application improve productivity, but beware of Baumol’s disease [8]. Some scholars have also used empirical data on the use of robots in developed countries such as the United States, the European Union, and Japan, and found that in these developed countries, the use of robots has promoted TFP [9–12].

Export activity boosted labor productivity significantly in the short term, while processing trade and low-tech exports inhibited the improvement of labor productivity by corporate exports [13]. Export firms with lower productivity and higher financing constraints tend to select processing trade [14]. Tao et al., find that firms of different trade types indeed show systematic differences in export participation: improvement in productivity significantly constrains exports of firms engaged only in processing trade, promotes exports of firms engaged in ordinary trade and has no significant effect on trade of firms engaged in both processing and ordinary trades [15]. Jin & Hu found that enterprises can alleviate financing constraints by expanding exports, but subject to productivity, lower-productivity enterprises can only expand exports through processing trade, while higher-productivity enterprises can expand exports through general trade [16]. Lu et al., find that the effect of productivity on the general and processing trade exports of enterprises is completely different [17]. The increase of TFP will increase the general trade export tendency of enterprises, while in the export decision of processing trade, productivity and enterprises export tendency are negative. In conclusion, a large number of empirical studies have shown that productivity improvement has an inhibitory effect on processing trade exports, while promoting general trade exports.

### The use of industrial robots, industrial upgrading, and the transformation of trade modes

Tang, find that the improvement of the regional industrial intelligence level can effectively strengthen the positive impact of regional innovation on industrial structure upgrade, with the characteristics of regional heterogeneity [18]. Previous studies have divided the mechanism of industrial intelligence on the upgrading of industrial structure into three aspects: factor substitution [19], technological innovation and cost reduction. At present, there are relatively many studies on artificial intelligence and industrial upgrading at home and abroad. Industrial robots integrate a large number of artificial intelligence technologies and are an important branch in the field of artificial intelligence. Guo & Hu find that AI has a significant positive effect on industrial structure upgrading [20]. Graetz & Michaels believes that the integration of robots and traditional industries can effectively improve the productivity of traditional sectors, realize the optimal allocation of production factors, and then promote the upgrading of industrial structure [21]. Guo pointed out that artificial intelligence can promote the upgrading of traditional industries through scale and competition effects based on a multi-sector dynamic general equilibrium model [22]. Hu & Du believe that intelligence can significantly improve productivity, thereby promoting the optimization and upgrading of industrial structure [23].

Ni selected panel data from 28 provinces in China from 2004 to 2015 to conduct an empirical analysis, and found that industrial upgrading as a whole promoted the growth of China’s general trade exports and the transformation and optimization of the structure of export trade [24]. Assche and Biesebroeck believe that the transformation and upgrading of China’s processing trade exports are mainly achieved through industrial upgrading, quality upgrading or product upgrading, and functional upgrading [25]. And enterprise innovation is conducive to the increase of the proportion of general trade, that is, to promote the transformation and upgrading of enterprises export patterns [26]. Cui & Chen analyzed the R&D expenditure and other factors on the impact of China’s general trade and found that the increase in R&D expenditure promotes general trade export [27]. In essence, industrial robots are a new technology with a wider range and deeper impact as well. As computers can imitate the human brain, machines can not only continue repetitive and simple labor as is in the past, but also play more complex social roles and even work beyond human physical and cognitive ability. Therefore, its impact on general and processing trade will be expanded.

### The use of industrial robots, manufacturing employment and the transformation of trade modes

There are two different views from past research on the effects of robot adoption in labor market. The first view favors that the application of industrial robots mainly exerts the employment substitution effect, suggesting that an increased robot use may bring down the demand for labor by replacing low-skilled work entirely and high-skilled work partially under certain conditions [28–34]. By contrast, the second view argues for employment creation effect, wherein there may be increase in the demand for labor in industries or tasks that arise as a result of technological advances associated with robot adoption [35–38]. In addition, some scholars believe that the impact of industrial robot applications on workers with different skills is nonlinear [39–41]. In a word, the application of industrial robots changes the employment structure through the dual effects of substitution and creation, which is ultimately manifested in the increase in the employment rate of the service industry in various countries.

In addition, there is a positive correlation between the proportions of manufacturing employment and processing trade in export, with the latter greatly dependent on employment in manufacturing industry. Through an empirical study based on panel data of manufacturing and its sub-industries from 28 provinces from 2006 to 2013, Ma & Liu arrives at the conclusions as follows: the processing trade proportion, which stands for low-end and labor-intensive production mode, has a significantly positive relation with local manufacturing employment proportion [42]. According to the panel data from 28 provinces from 2009 to 2016, Lu found that the proportion of processing trade in each province is positively related to the that of local manufacturing employment [43].

**Fig 2:**
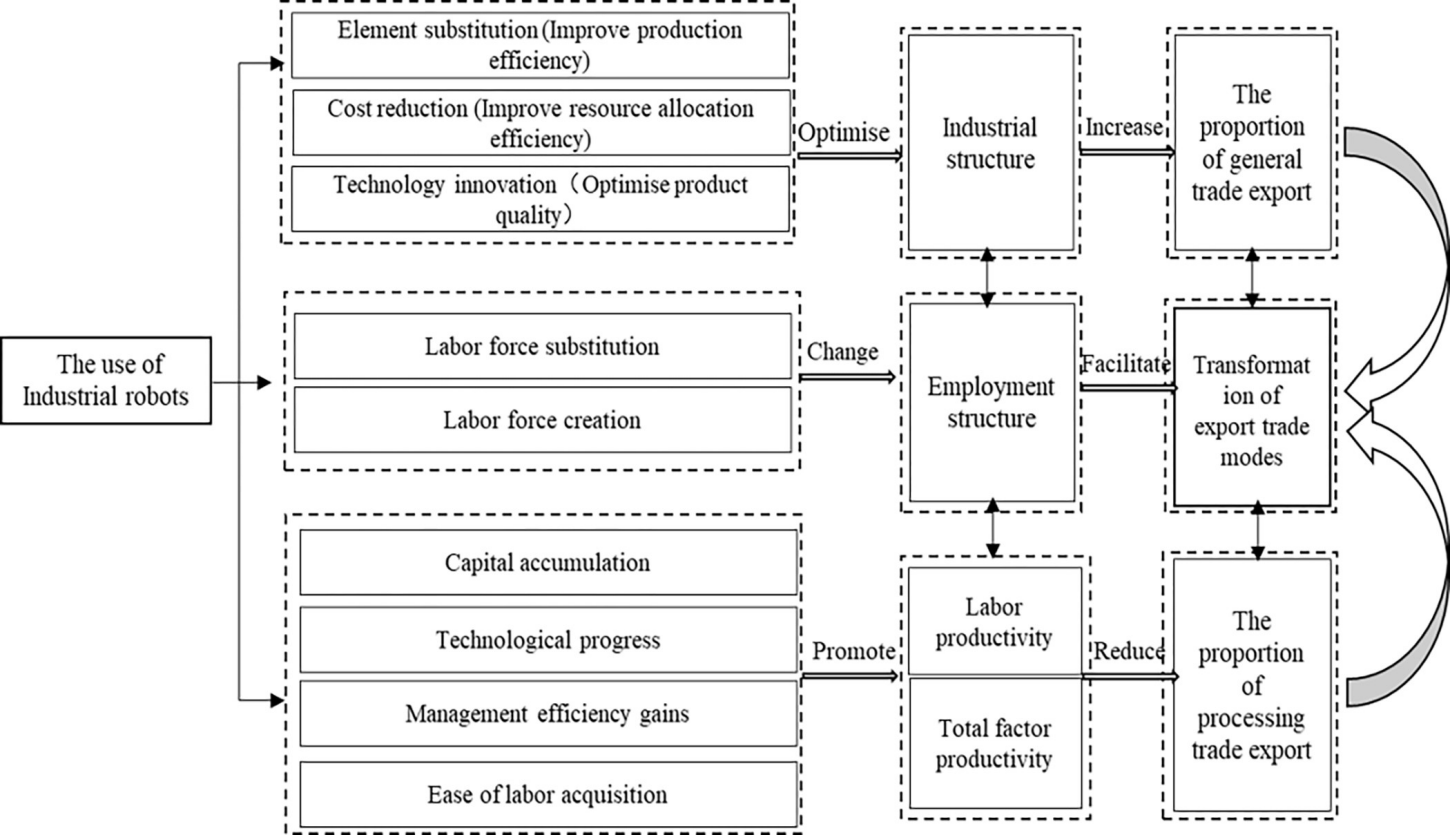
Mechanism’s diagram of the use of industrial robots promotes the transformation of trade patterns.

Fig 2 shows the relationship between the application of industrial robots and the transformation of export trade mode. On this basis, we put forward the following three hypotheses:

**Hypothesis 1:** The use of industrial robots will reduce the proportion of processing trade.
**Hypothesis 2:** The use of industrial robots will increase the proportion of general trade.
**Hypothesis 3:** The use of industrial robots will promote the transition of processing trade to general trade (the structural transformation & upgrade of export trade).

## Methodology and data

### Model specification

Following Autor & Dorn [44] and Acemoglu & Restrepo [10], we construct the following econometric model to investigate the impact of industrial robot application on processing trade based on hypothesis 1:

$${\mathrm{process}}_{it}=\alpha +\beta_{1}*{\mathrm{robit}}_{it}+X\gamma +\mu_{i}+v_{t}+\zeta_{it}$$ (1)

According to hypothesis 2, we construct the following econometric model to investigate the impact of industrial robot application on general trade:

$${\mathrm{general}}_{it}=\alpha +\beta_{2}*{\mathrm{robit}}_{it}+X\gamma +\mu_{i}+v_{t}+\zeta_{it}$$ (2)

According to hypothesis 3, we construct the following econometric model to investigate the impact of industrial robot application on trade pattern transformation:

$${\mathrm{trans}}_{it}=\alpha +\beta_{3}*{\mathrm{robit}}_{it}+X\gamma +\mu_{i}+v_{t}+\zeta_{it}$$ (3)

where subscripts *i* and *t* represent province and time respectively; *process*, *general*, *trans* is the dependent variables, representing measurable indicators of processing trade, general trade and trade mode transformation respectively; *robot* is the key explanatory variable, representing the penetration of industrial robot; ***X*** is other control variables that affect the key variable; *γ* is the corresponding coefficient matrix of control variables; *μ* and *ν* are province fixed effect and year fixed effect respectively, indicating unobservable factors affected by region and time; *ζ*_*it*_ is the random disturbance term. *ζ*_*it*_ is the random disturbance term; *β* is the coefficient of our concern, where if *β*_1_<0, it shows that the use of industrial robots will reduce the proportion of processing trade, then the Hypothesis 1 can be verified; and if *β*_2_>0, it indicates that the use of industrial robots will increase the proportion of general trade, and the Hypothesis 2 can be verified; if *β*_3_>0, it shows that the use of industrial robots will promote the transition of processing trade to general trade, and the Hypothesis 3 can be verified.

### Variables and data

#### Explained variables

(1) Proportion of processing trade (*process*): measured by the share of processing trade exports in total exports. Liu and Zhang used the ratio to measure the transformation of export modes, where a decline in the proportion of processing trade indicates the transformation and upgrading of enterprise [45].

(2) Proportion of general trade (*general*): measured by the share of general trade exports in total exports. Brandt & Morrow used the ratio to measure the transformation of enterprise export trade, where the increase in the share of general trade shows the transformation and upgrading of enterprise export trade [46].

(3) The transformation of trade modes (*trans*): measured by the ratio of general trade exports to processing trade exports. Fu & Lu used the ratio to measure the transformation of export modes. General trade and processing trade are positively related to economic growth, but the elasticity coefficient of general trade is almost twice more than that of processing trade. The expected symbol of *β*_3_ is positive.

#### Key explanatory variables

Drawing on existing research [47], we choose the exposure to robots as the key explanatory variable to conduct empirical research. Exposure to robots is another word for robot density; it measures the distribution density and utilization of industrial robots in a certain area, that is, robot numbers owned by per thousand workers. First, we suppose that the distribution of industrial robots in a certain industry is consistent in all regions of the country, then the density of industrial robots in a certain region depends on the share of employment in all industries of the region. When calculating the regional robot density, we take the share of employment in various industries in the region as the weight, and add up the robot density in all industries, so that the penetration rate of industrial robots can be obtained as follows:

$$robot=\sum_{i=1}^{n}l_{si}^{t}\times R_{i}^{t}/L_{i}^{t}$$ (4)

where $l_{si}^{t}=L_{si}^{t}/L_{s}^{t}$ is the weight, referring to the employment share of industry *i* in province *s* at year *t*. $L_{si}^{t}$ is the total employment of industry *i* in province *s* at year *t*. $L_{s}^{t}$ is the total employment of province *s* in year *t*. $L_{i}^{t}$ is the total employment of industry *i* at the national level in year *t*. $R_{i}^{t}$ is the quantity of industrial robot of industry *i* at the national level in year *t*, then $\frac{R_{i}^{t}}{L_{i}^{t}}$ refers to the density of industrial robots of industry *i* at the national level in year *t*. By summing up the robot density of all relevant industries in all provinces, the penetration rate of industrial robots can be obtained. We use the number of newly installed and the stock of industrial robots to measure the penetration rate of industrial robots, which are expressed in robot1 and robot2 respectively.

#### Controlled variables

Combining the analysis of theoretical part, and considering the robustness of empirical results, we mainly choose the following indicators as control variables:

Economic development level (*lnpgdp*): following Graetz & Michaels [21], we use the log value of per capita GDP to measure it.

Urbanization rate (*urb*): measured by the proportion of urban residents in total population. Urbanization can affect general trade and processing trade through the transfer of production factors and industrial agglomeration [48].

Investment level in fixed assets (*invent*): measured by the total investment in fixed assets in proportion of GDP. Fixed asset investment and infrastructure improvement can significantly promote export trade [49].

Financial level (*fin*): measured by the ratio of financial revenue to expenditure. The financial level affects the transformation and upgrading of processing trade through industrial support ability [50].

Environment pressure (*evn_inv*): the completed investment volume in industrial pollution control (billions of yuan) as a proportion of GDP. Various inputs to control environmental pollution show a significant negative correlation with the volume of exports [51].

Human capital attainment (*human*): measured by the number of college students per hundred people. Ke used the number of college students per ten thousand people as a measurement index [52].

The level of opening (*fdi*): measured by the ratio of foreign direct investment tax to GDP. Opening-up policy will affect the export trade structure through technology spillover effect and other channels [53].

#### Data sources

There are three main sources of data in this section. First, the data of industrial robots comes from the International Federation of Robotics (IFR), which statistically shows the use status of industrial robots in each industry of each country. It is mainly where we draw the new installation and stock data of China’s sub-industry. Second, the employment data of provincial sub-industry mainly come from the *China Labor Statistics Yearbook* and the *China Industrial Economic Statistics Yearbook*. Third, the data of export trade patterns. The statistical yearbook proves some but yet to be completed. We mainly use customs data to identify the total amount of each export trade mode in each province every year, and then calculate the proportion of processing trade and general trade. As the latest customs data is from 2000 to 2019, while the industry data of China’s industrial robot is available since 2006, we choose 2006–2019 as the research time period and 31 provincial administrative regions as research districts (excluding Hong Kong, Macao and Taiwan), so the final sample number is 434. Table 1 provides descriptive statistics of the main variables.

**Table 1:** Descriptive statistics of variables.

Variables	Obs	Mean	Std. Dev.	Min	Max
Explained Variable					
process	434	0.596	0.231	0.038	1.000
General	434	0.268	0.194	0.000	0.774
Trans	434	-1.162	1.353	-4.605	1.300
Core Explanatory Variables					
robot1	434	0.826	0.922	0.001	4.493
robot2	434	3.254	4.459	0.001	31.369
Control Variables					
Lnpgdp	434	10.512	0.613	8.663	12.009
Urb	434	0.537	0.143	0.211	0.896
Invent	434	0.743	0.269	0.073	1.720
Fin	434	0.496	0.205	0.064	0.951
Evn_inv	434	0.140	0.130	0.007	0.986
Human	434	1.796	0.577	0.600	3.534
Fdi	434	0.393	0.506	0.047	5.818

Data sources: Compiled by the author based on relevant data.

## Results and discussion

### Benchmark regression

Table 2 reports the baseline regression results. Here we use the number of new industrial robots for measurement. Columns (1) and (2) consider the impact of industrial robot application on processing trade. Column (1) controls the region fixed effect and the year fixed effect, which alleviates the endogenous issues caused by time and urban character. The estimation show that the coefficient of industrial robot is significantly negative at the level of 1%, indicating that the use of industrial robots will significantly decrease the proportion of processing trade, which is consistent with Hypothesis 1. Column (2) introduces control variables, where the results are significant at the level of 5%. The estimation show that the coefficient of industrial robot rises from -0.028 to -0.039, suggesting that with the increase of control factors in the model, the impact of industrial robot application on processing trade has deepened. Columns (3) and (4) explore the impact of industrial robot application on general trade. Column (3) controls region and year fixed effect, and the estimation show that the coefficient of industrial robot is significantly positive at the level of 5%, indicating that the use of industrial robots has a catalytic effect on general trade, hence hypothesis 2 is verified. The result in column (4) shows that the interpretation ability of the model is dramatically enhanced after the introduction of control variables. Columns (5) and (6) investigate the impact of industrial robots’ application on the transformation of export. Column (5) controls the region and year fixed effect, and the estimation show that the coefficient of industrial robot is significantly positive at the level of 10%. Column (5) introduces control variables, where the result shows that the coefficient of industrial robot increased from 0.159 to 0.285, and the significant level also increased from 10% to 1%, indicating that the use of industrial robots has deepened its contribution to the transformation of exports after controlling other influencing factors. Here, hypothesis 3 is verified.

**Table 2:** Benchmark regression results.

	(1)	(2)	(3)	(4)	(5)	(6)
	process	process	general	general	trans	trans
robot1	-0.028***	-0.039**	0.030**	0.054***	0.159*	0.285***
	(0.006)	(0.015)	(0.015)	(0.015)	(0.089)	(0.088)
lnpgdp		-0.005		-0.054		-0.176
		(0.059)		(0.057)		(0.339)
urb		-0.861**		1.141***		4.720**
		(0.369)		(0.359)		(2.135)
inv_gdp		0.030		-0.009		-0.004
		(0.043)		(0.042)		(0.248)
fin		-0.282*		0.281*		1.209
		(0.168)		(0.164)		(0.974)
evn_inv		0.164**		-0.174***		-1.076***
		(0.063)		(0.062)		(0.366)
stu		-0.254***		0.227***		1.285***
		(0.042)		(0.041)		(0.241)
fdi		0.012		0.017		0.022
		(0.016)		(0.016)		(0.092)
_cons	0.619***	1.692***	0.243***	-0.344	-1.293***	-4.844
	(0.008)	(0.525)	(0.014)	(0.511)	(0.080)	(3.039)
*N*	434	434	434	434	434	434
*R* ^2^	0.773	0.845	0.723	0.792	0.807	0.849
F	19.360	16.994	3.920	16.659	3.170	13.561
Year FE	Y	Y	Y	Y	Y	Y
Province FE	Y	Y	Y	Y	Y	Y

*Notes*: The numbers in parenthesis are robust standard errors; P value are in square bracket

*, **, and *** represent 10%, 5%, and 1% significant level, respectively.

In terms of control variables, the impact of economic development level on the transformation of processing trade, general trade and trade mode is not significant, which may be resulted from the large differences in the level of economic development in different regions of China. The influence coefficient of urbanization on processing trade is significantly negative; on general trade, significantly positive; and on the transformation of trade mode, significantly positive. It indicates that the level of urbanization can promote the transformation of processing trade to general trade, which can optimize the export trade structure to a certain extent. The impact coefficient of human capital on processing trade is significantly negative; on general trade, significantly positive; and on trade transformation, significantly positive. It demonstrates that the richer human capital is, the more favorable it is for the transformation of processing trade to general trade, which will promote the structural transformation and upgrading of export trade. The impact coefficient of environmental pressure on export trade is significantly positive; on general trade, significantly negative; and on trade transformation, significantly negative. It shows that it has an inhibitory effect on the transition of processing trade to general trade, which may be because processing trade is stronger than general trade in terms of environmental protection, environmental standards and energy consumption standards [54].

### Robustness check

In order to prove the reliability of the above analysis results, we use the stock number of industrial robots as an alternative variable to test the robustness of the regression equation. Table 3 shows the estimation result of robustness check. Columns (1) and (2) consider the impact of industrial robot application on processing trade. The results show that the coefficient of industrial robot is significantly negative after controlling region and year fixed effect. Meanwhile, when the control variable is introduced, the coefficient increases from -0.004 to -0.005. Therefore, we can infer that the use of industrial robots will significantly decrease the proportion of processing trade. Compared with the first two columns in Table 2, the coefficient has decreased, yet the results are still significant. Columns (3) and (4) consider the impact of industrial robots’ application on general trade. The estimation results show that after controlling region and year fixed effect, whether the control variable is introduced or not, the coefficients are significantly positive at the level of 1%, demonstrating that the use of industrial robots will significantly increase the proportion of general trade. Columns (5) and (6) explore the impact of industrial robots on the transformation of export trade. The results show that while controlling region and year fixed effect, the coefficient is significantly positive; and the coefficient decreases from 0.197 to 0.035 after introducing the control variables, implying that after controlling other influencing factors, the contribution of the industrial robot’s application to the transformation of export, although decreases, still exists. The results are robust and reliable.

**Table 3:** Robustness test results.

	(1)	(2)	(3)	(4)	(5)	(6)
	process	process	general	general	trans	trans
robot2	-0.004***	-0.005*	0.025***	0.007***	0.197***	0.035**
	(0.001)	(0.003)	(0.004)	(0.003)	(0.027)	(0.016)
lnpgdp		-0.019		-0.035		-0.074
		(0.058)		(0.057)		(0.339)
urb		-0.802**		1.067***		4.291**
		(0.370)		(0.361)		(2.143)
inv_gdp		0.049		-0.033		-0.141
		(0.042)		(0.041)		(0.242)
fin		-0.283*		0.284*		1.217
		(0.169)		(0.165)		(0.982)
evn_inv		0.162**		-0.173***		-1.066***
		(0.064)		(0.062)		(0.369)
stu		-0.256***		0.229***		1.296***
		(0.042)		(0.041)		(0.243)
fdi		0.011		0.018		0.031
		(0.016)		(0.016)		(0.093)
_cons	0.610***	1.781***	0.187***	-0.468	-1.801***	-5.492*
	(0.007)	(0.527)	(0.016)	(0.514)	(0.107)	(3.056)
*N*	434	434	434	434	434	434
*R* ^2^	0.768	0.843	0.098	0.789	0.122	0.846
F	11.097	16.419	40.871	15.675	53.505	12.707
Year FE	Y	Y	Y	Y	Y	Y
Province FE	Y	Y	Y	Y	Y	Y

*Notes*: The numbers in parenthesis are robust standard errors; P value are in square bracket

*, **, and *** represent 10%, 5%, and 1% significant level, respectively.

#### Endogenous problems

The previous research verifies that the use of industrial robots is conducive to the transformation of export trade patterns. However, there is an issue of reverse causality, and the way of trade may also affect the use of robots. Whether a large number of industrial robots need to be invested may be related to the labor structure of export trade and general trade themselves. The transformation of export trade mode may lead to changes in labor structure, which in turn affects the scale of the use of industrial robots. Bidirectional causality may exist between them, which may lead to bias in quantitative regression results. Therefore, in order to solve the endogenous problem caused by possible reverse causality, this paper adopts lag one phase of industrial intelligence to re-estimate.

#### Lag one phases test

Table 4 reports the result of the lag one phase reassessment of industrial robots. The estimated results in the first three columns show that after controlling the two fixed effect and other control variables, the impact coefficient of industrial robots on processing trade is negative; on general trade, positive; and on the transformation of trade pattern, also positive, with the data being -0.052,0.057,0.259 respectively, all significant at the level of 1%. Therefore, we can infer that the use of industrial robots has a significant lag effect on the transition of processing trade to general trade. In order to verify the robustness of the results, when the last three columns replace the core explanatory variables with the inventory of industrial robots, the regression results are still significant. It proves that the result is robust.

**Table 4:** Results of lag one phases test.

	(1)	(2)	(3)	(4)	(5)	(6)
	process	general	trans	process	general	trans
L.robot1	-0.052***	0.057***	0.259***			
	(0.016)	(0.016)	(0.095)			
L.robot2				-0.010***	0.011***	0.046**
				(0.004)	(0.004)	(0.021)
lnpgdp	0.045	-0.097*	-0.335	0.032	-0.082	-0.264
	(0.058)	(0.058)	(0.344)	(0.058)	(0.058)	(0.343)
urb	-0.995***	1.236***	5.442**	-0.948**	1.185***	5.172**
	(0.377)	(0.377)	(2.225)	(0.378)	(0.379)	(2.230)
inv_gdp	0.040	-0.036	-0.119	0.055	-0.052	-0.197
	(0.040)	(0.040)	(0.238)	(0.040)	(0.040)	(0.234)
fin	-0.574***	0.528***	2.435**	-0.576***	0.530***	2.428**
	(0.180)	(0.180)	(1.064)	(0.182)	(0.182)	(1.073)
evn_inv	0.208***	-0.224***	-1.189***	0.206***	-0.222***	-1.176***
	(0.065)	(0.065)	(0.382)	(0.065)	(0.065)	(0.384)
stu	-0.260***	0.241***	1.338***	-0.261***	0.242***	1.342***
	(0.043)	(0.043)	(0.255)	(0.043)	(0.043)	(0.256)
fdi	0.023	0.010	-0.004	0.022	0.012	0.004
	(0.015)	(0.015)	(0.089)	(0.015)	(0.015)	(0.089)
_cons	1.382***	-0.065	-4.153	1.475***	-0.168	-4.633
	(0.525)	(0.525)	(3.100)	(0.526)	(0.526)	(3.102)
*N*	403	403	403	403	403	403
*R* ^2^	0.868	0.814	0.869	0.867	0.812	0.868
F	19.342	17.462	13.933	18.743	16.756	13.472
Year FE	Y	Y	Y	Y	Y	Y
Province FE	Y	Y	Y	Y	Y	Y

*Notes*: The numbers in parenthesis are robust standard errors; P value are in square bracket

*, **, and *** represent 10%, 5%, and 1% significant level, respectively.

#### Instrumental variable estimate

Previous section adopts lag one phase method, while more is needed to address the endogenous problems thoroughly, which mainly originate from the following two aspects. First is the problem of missing variables. Although the benchmark empirical model has controlled the influencing factors that may lead to the transition of processing trade to general trade in terms of human capital level, urbanization, openness and so on, the omission of variables may still cause endogenous problems due to the availability of variables. For another, there may be a reverse causal relationship between the use of industrial robots and the transformation of export trade patterns. The transformation of export trade mode may lead to changes in the labor structure, which in turn affects the scale of the use of industrial robots. Therefore, this paper attempts to reassess the model by finding instrumental variables to solve potential endogenous problems.

In the existing relevant literature, Graetz & Michaels [21] adopted two instrumental variables to analyze the economic impact of industrial robots in 17 countries from 1993–2007. The first instrument for robot densification is an industry-level measure that they call “replaceability”, which was a construct based on the data from IFR on robot applications, the U.S. Census occupational classifications, and the distribution of hours across occupations and industries from the 1980 U.S. Census. The second is whether the industry needed to use robotics arms during the period of analysis. Robotic arms are a technological characteristic of robots that comes from the supply side and are not driven by demand-side factors that may reflect reverse causality. Their research subjects, however, are from developed countries, so there are essential structural differences in China in its use of industrial robots in manufacturing industry. Coupled with the lack of relevant industry data, these two instrumental variables are not applicable to China’s situation. Acemoglu & Restrepo analyzed the effect of the increase in industrial robot use between 1990 and 2007 on the US labor markets, using industrial robot installations in Germany, Japan and South Korea as an instrumental variable for that in the US. Due to international competitions among several major manufacturing countries, countries have shown a high convergence in the application scale of new production technologies and equipment.

Table 5 presents the estimated outcomes of tool variables. We reevaluated robot2 as a substitute variable to improve the robustness of instrumental variable regression results, The first stage estimate shows that the regression coefficients of robot1 and robot2 are positive at the level of 1%, indicating that the industrial robot’s adoption in the US will significantly improve the process of industrial intelligence in China. Furthermore, the Cragg-Donald Wald F statistics are much higher than 16.83 (the critical value of weak IV above the significant level of 10%). As a result, the possibility of weak IV can be ruled out [55]. The instrumental variable we chose has a high correlation with the original explanatory variable. Table 5 shows that Anderson canon. corr. LM statistic strongly rejects the original hypothesis of instrumental variable under identification. Therefore, there is no under-identified problem with instrumental variables we chose. In conclusion, the choice of instrumental variables in this paper is reasonable and effective, and it can help to solve potential endogenous problems.

**Table 5:** Results of instrumental variable estimate.

	(1)	(2)	(3)	(4)	(5)	(6)
	process	general	trans	process	general	trans
robot1	-0.021***	0.053*	0.282*			
	(0.008)	(0.028)	(0.146)			
robot2				-0.005*	0.009***	0.044**
				(0.003)	(0.003)	(0.019)
lnpgdp	-0.014	-0.053	-2.081***	-0.019	-0.037	-0.084
	(0.060)	(0.058)	(0.253)	(0.059)	(0.057)	(0.339)
urb	-0.796**	1.137***	0.193	-0.812**	1.098***	4.444**
	(0.379)	(0.368)	(0.920)	(0.371)	(0.362)	(2.151)
inv_gdp	0.049	-0.010	0.079	0.047	-0.025	-0.103
	(0.049)	(0.048)	(0.294)	(0.042)	(0.041)	(0.246)
fin	-0.275	0.281*	5.373***	-0.285*	0.290*	1.247
	(0.169)	(0.164)	(0.447)	(0.169)	(0.165)	(0.983)
evn_inv	0.160**	-0.174***	-0.887**	0.163**	-0.176***	-1.081***
	(0.064)	(0.062)	(0.368)	(0.064)	(0.062)	(0.369)
stu	-0.258***	0.227***	1.146***	-0.255***	0.226***	1.283***
	(0.042)	(0.041)	(0.129)	(0.042)	(0.041)	(0.244)
fdi	0.011	0.017	0.273***	0.011	0.018	0.028
	(0.016)	(0.016)	(0.104)	(0.016)	(0.016)	(0.093)
*N*	434	434	434	434	434	434
*R* ^2^	0.260	0.259	0.600	0.256	0.246	0.209
F	16.178	15.465	76.613	16.373	15.755	12.765
Year FE	Y	Y	Y	Y	Y	Y
Province FE	Y	Y	Y	Y	Y	Y
First stage						
robot1_IV	1.826***					
	(0. 146)					
robot2_IV				1.817***		
				(0.061)		
Cragg-Donald Wald F statistic		155.78 [0.000]			862.66 [0.000]	
Anderson canon. corr. LM statistic		125.72 [0.000]			300.80 [0.000]	

*Notes*: The numbers in parenthesis are robust standard errors; P value are in square bracket

*, **, and *** represent 10%, 5%, and 1% significant level, respectively.

After solving the potential endogenous problems, the use of industrial robots continue to have a significant impact on the transformation of China’s processing trade to general. For every 1% increase in the number of newly installed industrial robots, the proportions of processing trade in China’s export trade decreased by 2.1%, of general trade increased by 5.3%, and the ratio of general trade to export trade increased by 28.2%. In addition. For every 1% increase in the stock of industrial robots, the proportions of processing trade decreased by 0.5%, of general trade increased by 0.9%, and the ratio of general trade to export trade increased by 4.4%. Meanwhile, the estimated results closely match our baseline estimates.

### Mechanism analysis

There are remarkable regional differences in China because of its vast territory and unbalanced economic development, so the development of industrial robots varies significantly among regions, leaving heterogeneous influence on exports. According to China’s traditional geographical divisions and economic development levels, we chose 31 provincial level administrative districts in mainland as geographic units and further divide them into eastern, central and western areas. The eastern area includes Beijing, Tianjin, Hebei, Liaoning, Shanghai, Jiangsu, Zhejiang, Fujian, Shandong, Guangdong and Hainan; the central includes Shanxi, Jilin, Heilongjiang, Anhui, Jiangxi, Henan, Hubei and Hunan; and the rest 12 regions for the western area.

Table 6 presents the estimation outcomes for the above three regions. It shows that the use of industrial robots has played a significant role in promoting the transformation of processing to general trade in different areas with differences in coefficients. Among them, the number of newly installed industrial robots has the most significant effect on the transformation of processing trade to general trade in western region. Specifically, for one-percentage point increase in the number of newly installed industrial robots, the proportions decreased by 10.2% in processing trade and increased by 8.9% in general trade, while the ratio of general trade to processing trade increased by 54.3%, all of which are significant at the level of 1%. The reason is that human capital and productivity level in western region is much lower than those of in eastern region. The improvement of industrial intelligence will significantly strengthen local automated and intelligent production processes, upgrade the employment structure, and promote the transformation and upgrading of processing trade to general trade. In addition, the stock of industrial robots has the most significant impact on export trade in the central region. For every 1% increase in the stock of industrial robots, the proportions decreased by 1.6% in processing trade and increased by 2.2% in general trade, while the ratio of general trade to processing trade increased by 9.1%, all of which are significant at the level of 5%. This implies regional heterogeneity in the use of industrial robots in promoting the transition of processing trade to general trade.

**Table 6:** Regression results for eastern, middle and western China.

Part I: Eastern Region	process	general	trans	process	general	trans
	(1)	(2)	(3)	(4)	(5)	(6)
robot1	-0.035**	0.147***	0.154***			
	(0.015)	(0.029)	(0.046)			
robot2				-0.006***	0.005**	0.066***
				(0.002)	(0.002)	(0.024)
*N*	154	154	154	154	154	154
*R* ^2^	0.898	0.914	0.924	0.875	0.900	0.931
F	9.269	2.756	27.534	10.083	30.664	5.757
Part II: middle Region	process	general	trans	process	general	trans
	(1)	(2)	(3)	(4)	(5)	(6)
robot1	-0.030***	0.050*	0.402***			
	(0.011)	(0.027)	(0.050)			
robot2				-0.016**	0.022**	0.091**
				(0.008)	(0.008)	(0.041)
*N*	112	112	112	112	112	112
*R* ^2^	0.770	0.799	0.745	0.770	0.803	0.752
F	8.457	9.233	7.188	8.510	9.568	7.680
Part III: Western Region	process	general	trans	process	general	trans
	(1)	(2)	(3)	(4)	(5)	(6)
robot1	-0.102***	0.089***	0.543***			
	(0.020)	(0.019)	(0.140)			
robot2				-0.011***	0.009***	0.058**
				(0.004)	(0.003)	(0.024)
*N*	168	168	168	168	168	168
*R* ^2^	0.918	0.854	0.871	0.909	0.839	0.863
F	10.726	10.438	7.788	8.018	7.846	6.250
Control Variables	Y	Y	Y	Y	Y	Y
Year FE	Y	Y	Y	Y	Y	Y
Province FE	Y	Y	Y	Y	Y	Y

*Notes*: The numbers in parenthesis are robust standard errors; P value are in square bracket

*, **, and *** represent 10%, 5%, and 1% significant level, respectively.

## Conclusions and policy implication

The impact of a large-scale application of industrial robots on China’s social economy catches increasing attention and research from both academia and government departments. This article is based on the sub-industry data provided by the Industrial Robot Alliance. the employment data provided by the China Labor Statistics Yearbook and China Industrial Economic Statistics Yearbook, and the processing trade and export trade data provided by the customs database. The paper matched the panel data from 2006–2019 on the use of industrial robots and export trade in 434 prefecture-level cities in the year to examine the impact of industrial robot use on the transformation of export trade. The conclusions are as follows:(1) The use of industrial robots has significantly suppressed the export share of processing trade, increased the share of general trade exports, and promoted the transformation and upgrading of the export model of Chinese enterprises, while the potential endogenous problems were addressed by replacing explanatory variables and instrumental variable estimation methods, rendering the aforementioned conclusion robust and credible. (2) The use of industrial robots has a significant lagging effect on the transformation of processing trade to general trade. After considering the delayed reaction of the trade mode structure, the promotion effect of industrial robots on the transformation and upgrading of China’s export trade mode continues to be significant. (3) The influence of industrial robots’ adoption on export trade patterns reveals significant regional differences. The number of newly installed industrial robots has a more pronounced effect on the trade transformation in the western and central regions than that in the eastern region, while the stock of industrial robots is exported to the central region. (4) The promotion effect of industrial robots on general trade is greater than the restraint effect on processing trade.

From the research conclusions, we propose the following policy recommendations:

First, improve the technical level of industrial robots and actively promote the development and application of industrial robots. When the core technology of China’s manufacturing industry reaches a certain level, it will help transform China’s trade structure from an importer of intermediate products to an exporter of intermediate products. Furthermore, when China’s accumulated technology level is sufficient to reverse relative competitive advantages of foreign enterprises, the trade structure will naturally achieve transformation and upgrading. Therefore, improving the technical level of domestic industrial robots and the domestic factor endowment structure are essential for realizing a dynamic transformation of China’s comparative advantages and accelerating the transformation of trade patterns. In terms of policy, the government should not only increase fiscal and taxation support in the field of industrial intelligence, but also promote human capital accumulation, build talent teams, and encourage enterprises to develop industrial robot technologies beneficial to the transformation of trade methods. Specific policies include increasing the value-added tax deduction, or increasing the amount of financial subsidies for industrial intelligent patents such as industrial robots, etc.

Secondly, make use of the situation and follow the trend to guide foreign trade companies to transform and upgrade in the Industrial intelligence environment. Processing trade of the *low-end locking* value chain causes long dependence of Chinese enterprises’ key technology on external demand. Such a deeply rooted processing trade development model of "the international circulation" renders the enterprise susceptible to economic consequences of being “singled out” in export, harming China’s export trade stability and high-quality development. Relatively speaking, general trade is more involved in the "domestic cycle" and depends on both international and domestic markets, so it has a stronger risk tolerance when impacted by the uncertainty of external economic policies. The rising uncertainty of international economic policies has reduced the opportunity cost of enterprises’ transformation and upgrading. At this time, the government can utilize the situation to help enterprises achieve transformation and upgrading.

Thirdly, from the perspective of regional development, we should accelerate the integration of industrial robots in domestic manufacturing market, promote the cross-regional free flow of such factors among different regions and cities. Different regions in China must further reduce policy barriers, break down local protectionism, and increase local enterprises’ access to high-quality industrial robot at home and abroad. For the central and western regions, it is necessary to adopt market competition mechanism, further expand the opening of industrial robots, and accelerate the utilization of industrial robots in the central and western regions.

Fourthly, actively improve the industrial supporting capability by setting up a complete industrial robot production value chain, facilitating the extension of industry chain and value chain control. Affiliating industries are the foundation for export trades in promoting the growth of local industries and the upgrading and transformation of its industrial model. Because industrial robot has important effects on international competitiveness and national economic security and with a gradually higher permeability of overall economy, the shortage of a complete value chain and key link of the intelligent propulsion control will affect the whole industry. As far as this paper is concerned, it will endanger the transformation and upgrading of China’s export trade. The country should view the construction of industrial intelligent value chain from a strategic height of national economic security.

This paper examines the impact of industrial robot use on China’s trade structure from the perspective of trade mode transformation and draws some meaningful conclusions and policy implications. However, there are still some improvement directions in this paper. First of all, the transformation of trade structure has multiple dimensions, in addition to this article’ focus on ways of export trade, there are export products quality and trade pattern innovation that appears in enterprise value chain, such as different dimensions, which will be affected by industrial robots that deserves further research.
